# A nomenclature and classification for the congenital myasthenic syndromes: preparing for FAIR data in the genomic era

**DOI:** 10.1186/s13023-018-0955-7

**Published:** 2018-11-26

**Authors:** Rachel Thompson, Angela Abicht, David Beeson, Andrew G. Engel, Bruno Eymard, Emmanuel Maxime, Hanns Lochmüller

**Affiliations:** 1 Institute of Genetic Medicine, Newcastle University, Newcastle upon Tyne, UK; 2 Medical Genetics Centre, Munich, Germany; 3 Nuffield Department of Clinical Neurosciences, University of Oxford, Oxford, OX3 9DU UK; 4 Department of Neurology, Mayo Clinic, Rochester, USA; 5 Institut de Myologie, Paris, France; 6 INSERM US14 - Orphanet, Plateforme Maladies Rares, 75014 Paris, France; 7 Children’s Hospital of Eastern Ontario (CHEO) Research Institute, University of Ottawa, Ottawa, ON K1H 8L1 Canada; 8 Department of Neuropediatrics and Muscle Disorders, Medical Center – University of Freiburg, Faculty of Medicine, Freiburg, Germany; 9 Centro Nacional de Análisis Genómico (CNAG-CRG), Center for Genomic Regulation, Barcelona Institute of Science and Technology (BIST), Barcelona, Spain

**Keywords:** Congenital myasthenic syndromes, CMS, Neuromuscular junction, Neuromuscular disease, Nomenclature, Ontology, Nosology, Coding, Classification, Rare disease

## Abstract

**Background:**

Congenital myasthenic syndromes (CMS) are a heterogeneous group of inherited neuromuscular disorders sharing the common feature of fatigable weakness due to defective neuromuscular transmission. Despite rapidly increasing knowledge about the genetic origins, specific features and potential treatments for the known CMS entities, the lack of standardized classification at the most granular level has hindered the implementation of computer-based systems for knowledge capture and reuse. Where individual clinical or genetic entities do not exist in disease coding systems, they are often invisible in clinical records and inadequately annotated in information systems, and features that apply to one disease but not another cannot be adequately differentiated.

**Results:**

We created a detailed classification of all CMS disease entities suitable for use in clinical and genetic databases and decision support systems. To avoid conflict with existing coding systems as well as with expert-defined group-level classifications, we developed a collaboration with the Orphanet nomenclature for rare diseases, creating a clinically understandable name for each entity and placing it within a logical hierarchy that paves the way towards computer-aided clinical systems and improved knowledge bases for CMS that can adequately differentiate between types and ascribe relevant expert knowledge to each.

**Conclusions:**

We suggest that data science approaches can be used effectively in the clinical domain in a way that does not disrupt preexisting expert classification and that enhances the utility of existing coding systems. Our classification provides a comprehensive view of the individual CMS entities in a manner that supports differential diagnosis and understanding of the range and heterogeneity of the disease but that also enables robust computational coding and hierarchy for machine-readability. It can be extended as required in the light of future scientific advances, but already provides the starting point for the creation of FAIR (Findable, Accessible, Interoperable and Reusable) knowledge bases of data on the congenital myasthenic syndromes.

## Background

Congenital myasthenic syndromes (CMS) are rare inherited neuromuscular disorders characterized by fatigable weakness of skeletal muscle owing to compromised function of the neuromuscular junction (NMJ). First described in the 1940s [1] as a potential rare “familial” form of infantile myasthenia contrasting with the more common autoimmune-mediated myasthenia gravis, the first genetic defects associated with the condition were reported in the 1990s [2]. With the advent of next-generation sequencing (NGS), the number of genetic defects reported as causative of a CMS phenotype has increased dramatically, with as many as 31 genes now implicated [3]. The known types of CMS range in frequency from more than 1000 individuals to single sporadic reported cases, and around 20 to 40% of cases remain without a genetic diagnosis after exome sequencing [3]. Although all CMS share the common features of NMJ pathology and fatigable weakness, the severity of the disease, its course of progression, specific phenotypic manifestations and even effective treatments are highly variable between the different types. Furthermore, different pathogenic changes within the same gene may result in different pathological processes and therefore markedly different disease manifestations and therapeutic options [4].

Within this complex environment, it is clear not only that precision in diagnosis is important in order to correctly define the disease and institute appropriate treatment, but that precision in coding or classification of this diagnosis is a prerequisite for any attempt at systematizing knowledge and linking it to a specific CMS type. Yet coding and classification has long been a vexed issue in the rare disease field as a whole, going far beyond CMS [5]. Where clinical or genetic entities do not have a named entry in disease coding systems, they are often invisible in clinical records and inadequately annotated in information systems, since features that apply to one disease but not another cannot be adequately differentiated [6].

Coinciding with the dramatic increase in genomic data and computational approaches to diagnosis, recent years have seen the emergence of new data science approaches and their application to clinical problems to allow the systematization of existing and newly generated clinical knowledge in a way that is more accessible to computational analysis. This has been termed the FAIR data approach, an acronym that stands for Findable, Accessible, Interoperable and Reusable and represents the concept that the utility of clinical and research data is dramatically increased if it can be made accessible to reuse by others [7]. Precision in nomenclature terms is just one aspect of making a dataset FAIR, but nevertheless a crucial one in order to attach the right knowledge to the right disease. Our present study aimed to create a comprehensive classification for all CMS disease entities as a starting point that will then allow generation of FAIR-compliant datasets of knowledge about each type.

## Methods

We began by defining the CMS disease entities to be considered in the classification. We adopted a broad definition of CMS as any genetic neuromuscular condition manifesting with fatigable weakness of skeletal muscle and apparent NMJ involvement. We defined individual CMS “unique entities” at (a) gene level in cases where the presumed pathomechanism is identical for defects anywhere in a given gene, or (b) sub-gene level in cases where different defects in different regions of the same gene result in different disease manifestations due to differing pathomechanisms (e.g. to differentiate slow-channel from fast-channel syndromes within the same acetylcholine receptor gene). We did not split the classification to account for variable severity, age of onset or incomplete penetrance of phenotypic features where the underlying pathomechanism is the same, and we excluded non-CMS presentations of disorders caused by defects in the same genes that may also cause CMS presentation (e.g. kidney presentations of *LAMB2* defects). In the case of genetic entities affecting ubiquitous metabolic pathways (glycosylation defects, mitochondrial defects), some specific mutations cause a primary neuromuscular transmission defect, and these are included in our classification, while other mutations cause wider organ involvement, where the neuromuscular transmission defect may become irrelevant or not detectable (e.g. syndromic congenital disorders of glycosylation, encephalomyopathy), and these are then classified elsewhere.

The entities thus defined therefore aim to be those that from a data science perspective are sufficiently granular to allow the mapping of disease to feature and extend the range of knowledge about that specific disease entity. Based on this framework, through a literature review we developed a comprehensive listing of all unique CMS clinical and genetic entities described to date that met our criteria for inclusion. We captured the range of terminology used in the literature to describe or name each entity or group of entities. Using their publicly available online browsers, we then reviewed the most widely used medical and genetic coding systems to establish their coverage of these published CMS disease entities. Table 1 provides details of the coding systems analyzed, the browsers used and the summary results of the search.

**Table 1:** Coverage of congenital myasthenic syndromes by the major medical coding systems

Coding system	Terminology browser used	Coding for congenital myasthenic syndromes class/category	Coding for individual CMS types
International Classification of Disease (ICD) Revision 11	https://icd.who.int/browse11/l-m/en	8C61: Congenital myasthenic syndromes	No coding but textual description of four categories: Congenital myasthenic syndrome with presynaptic defect, Synaptic basal lamina-associated CMS, Congenital myasthenia with postsynaptic defect, CMS with glycosylation deficiency, Unidentified CMS.
International Classification of Disease (ICD) Revision 10	http://apps.who.int/classifications/icd10/browse/2016/en	G70.2: Congenital and developmental myasthenia	Not present
Medical Subject Headings (MeSH)	https://meshb.nlm.nih.gov/search	C16.320.590: Myasthenic Syndromes, Congenital	Not present
Systematized Nomenclature of Medicine – Clinical Terms (SNOMED CT)	http://browser.ihtsdotools.org/	230672006: Congenital myasthenia (disorder)	Not present
Orphanet nomenclature of rare diseases	https://www.orpha.net/consor/cgi-bin/Disease.php?lng=EN	ORPHA:590: Congenital myasthenic syndrome	Most granular level is absent. Subclasses are defined: Postsynaptic congenital myasthenic syndromes Presynaptic congenital myasthenic syndromes Synaptic congenital myasthenic syndromes Congenital myasthenic syndromes with glycosylation defect
Online Mendelian Inheritance in Man (OMIM)	http://omim.org/	N/A	Coding of 28 out of 39 entities with “phenotype MIM number” (for detail see Table 2) No hierarchies/ontological features

From the results of the initial stage of the research, we concluded that all existing coding systems had major gaps in coverage, in most cases caused by inadequate levels of granularity, with the most granular entities either completely or partially absent. Given the pressing need to define a fully granular classification for the “data science” purposes described above, we initiated a collaboration with Orphanet to extend the Orphanet nomenclature [8] to include our unique CMS disease entities. We aimed to avoid creation of a competing classification given the multiplicity of systems already in existence, and Orphanet was selected as the most suitable system for this collaboration because it aims to be a fully comprehensive coding system specifically designed for rare disease; it makes use of a hierarchical system or tree-like structure in which disease entities can be grouped in different logical ways; it includes mappings to many other coding systems at appropriate levels of granularity thus ensuring interoperability [9]; and it welcomes collaborations with domain experts for the purposes of extending its nomenclature. Orphanet has published a procedural document [10] for rare disease nomenclature in English that provides detailed guidance for naming entities, which states that names should be based on clinical practice, validated by experts in the field, comprehensive, consistent, and as stable as possible with regard to evolution of scientific knowledge. We therefore defined “descriptive names” for each entity in a manner consistent with the Orphanet guidelines, creating a clinically understandable name for each entity that should be stable notwithstanding the rapid advances in understanding the genetics of CMS. It is important to note that while the descriptive names are valuable from the perspective of human understanding, the essential point is that the disease entities are assigned unique identifiers within the coding system, which enables computer-readability and interoperability with other systems.

At the initial stage, the full listing of unique clinical entities that are classed as a CMS according to our definition is a non-hierarchical nosology or “flat” table (Table 2) mapped to the existing coding systems as appropriate. However, since Orphanet allows the creation of a hierarchical classification in which individual disorders may be grouped into one or multiple parent groups based on specific features, we also created an additional table in which we grouped all the unique entities from Table 2 based on etiological or other features (Table 3).

**Table 2:** Nomenclature proposals for individual CMS disease entities and mapping to pre-existing classifications

Gene involved	Proposed descriptive name	OMIM phenotype number and name	Treatment options [4, 11]	Existing Orphanet name (group level)	Names in literature (group level)	Names in literature (entity level)
*AGRN*	Congenital myasthenic syndrome due to agrin deficiency caused by pathogenic variants in *AGRN*	615120: Myasthenic syndrome, congenital, 8; CMS8 Alternative/former titles: Myasthenic syndrome, congenital, with pre- and postsynaptic defects; CMSPPD Myasthenic syndrome, congenital, due to agrin deficiency	Salbutamol or ephedrine as first line; avoid pyridostigmine / acetylcholinesterase inhibitors	ORPHA:98913 Postsynaptic congenital myasthenic syndromes AND ORPHA:98914 Presynaptic congenital myasthenic syndromes	• Defects in endplate development and maintenance	• Agrin deficiency
*ALG14*	Congenital myasthenic syndrome due to a defect of glycosylation caused by pathogenic variants in *ALG14*	616227: Myasthenic syndrome, congenital, 15; CMS15 Alternative/former titles: Myasthenic syndrome, congenital, without tubular aggregates; CMSWTA	Pyridostigmine as first line; may benefit from addition of 3,4-diaminopyridine	ORPHA:353327 Congenital myasthenic syndromes with glycosylation defect	• Limb-girdle-myasthenia with glycosylation deficiency • CMS due to abnormal glycosylation • Congenital defects of glycosylation • Defects in protein glycosylation	• ALG14 myasthenia
*ALG2*	Congenital myasthenic syndrome due to a defect of glycosylation caused by pathogenic variants in *ALG2*	616228: Myasthenic syndrome, congenital, 14; CMS14 Alternative/former titles: Myasthenic syndrome, congenital, with tubular aggregates 3; CMSTA3	Pyridostigmine as first line; may benefit from addition of 3,4-diaminopyridine	ORPHA:353327 Congenital myasthenic syndromes with glycosylation defect	• Limb-girdle-myasthenia with glycosylation deficiency • CMS due to abnormal glycosylation • Congenital defects of glycosylation • Defects in protein glycosylation	• ALG2 myasthenia
*CHAT*	Congenital myasthenic syndrome due to endplate choline acetyltransferase deficiency caused by pathogenic variants in *CHAT*	254210: Myasthenic syndrome, congenital, 6, presynaptic; CMS6 Alternative/former titles: Myasthenic syndrome, presynaptic, congenital, associated with episodic apnea; CMSEA Congenital myasthenic syndrome type Ia2, CMS1a2, CMS Ia2, Myasthenia, familial infantile, FIM, Myasthenia gravis, familial infantile, 2, FIMG2,	Pyridostigmine as first line; may benefit from addition of 3,4-diaminopyridine or salbutamol / ephedrine	ORPHA:98914 Presynaptic congenital myasthenic syndromes	• CMS with episodic apnea • Synthesis and Recycling of Acetylcholine	• Endplate choline acetyltransferase deficiency • CMS with episodic apnea
*CHRNA1*	Slow-channel congenital myasthenic syndrome due to an acetylcholine receptor defect caused by a pathogenic variant in *CHRNA1*	601462: Myasthenic syndrome, congenital, 1a, slow-channel; CMS1a Alternative/former titles: Myasthenic syndrome, congenital, type IIA, CMS2a, CMS 2a	Fluoxetine or quinidine as first line; avoid pyridostigmine / acetylcholinesterase inhibitors	ORPHA:98913 Postsynaptic congenital myasthenic syndromes	• Slow-channel syndrome, SCS • Kinetic abnormalities of the AChR	
*CHRNA1*	Fast-channel congenital myasthenic syndrome due to an acetylcholine receptor defect caused by pathogenic variants in *CHRNA1*	608930: Myasthenic syndrome, congenital, 1b, fast-channel; CMS1b Myasthenic syndrome, congenital, 1b, fast-channel; CMS1b	Pyridostigmine as first line; may benefit from addition of salbutamol / ephedrine or 3,4-diaminopyridine. Avoid β2-adrenergic agonists (fluoxetine / quinidine)	ORPHA:98913 Postsynaptic congenital myasthenic syndromes	• Fast-channel syndrome, FCS • Kinetic abnormalities of the AChR	
*CHRNA1*	Congenital myasthenic syndrome due to primary acetylcholine receptor deficiency caused by pathogenic variants in *CHRNA1*	N/A	Pyridostigmine as first line; may benefit from addition of 3,4-diaminopyridine or salbutamol / ephedrine	N/A	• Primary AChR deficiency	
*CHRNB1*	Slow-channel congenital myasthenic syndrome due to an acetylcholine receptor defect caused by a pathogenic variant in *CHRNB1*	616313: Myasthenic syndrome, congenital, 2a, slow-channel; CMS2a	Fluoxetine or quinidine as first line; avoid pyridostigmine / acetylcholinesterase inhibitors	ORPHA:98913 Postsynaptic congenital myasthenic syndromes	• Slow-channel syndrome, SCS • Kinetic abnormalities of the AChR	
*CHRNB1*	Fast-channel congenital myasthenic syndrome due to an acetylcholine receptor defect caused by pathogenic variants in *CHRNB1*	N/A	Pyridostigmine as first line; may benefit from addition of salbutamol / ephedrine or 3,4-diaminopyridine. Avoid β2-adrenergic agonists (fluoxetine / quinidine)	N/A	• Fast-channel syndrome, FCS • Kinetic abnormalities of the AChR	
*CHRNB1*	Congenital myasthenic syndrome due to primary acetylcholine receptor deficiency caused by pathogenic variants in *CHRNB1*	616314: Myasthenic syndrome, congenital, 2c, associated with acetylcholine receptor deficiency; CMS2c	Pyridostigmine as first line; may benefit from addition of 3,4-diaminopyridine or salbutamol / ephedrine	ORPHA:98913 Postsynaptic congenital myasthenic syndromes	• Primary AChR deficiency	
*CHRND*	Slow-channel congenital myasthenic syndrome due to an acetylcholine receptor defect caused by a pathogenic variant in *CHRND*	616321: Myasthenic syndrome, congenital, 3a, slow-channel; CMS3a	Fluoxetine or quinidine as first line; avoid pyridostigmine / acetylcholinesterase inhibitors	ORPHA:98913 Postsynaptic congenital myasthenic syndromes	• Slow-channel syndrome, SCS • Kinetic abnormalities of the AChR	
*CHRND*	Fast-channel congenital myasthenic syndrome due to an acetylcholine receptor defect caused by pathogenic variants in *CHRND*	616322: Myasthenic syndrome, congenital, 3b, fast-channel; CMS3b	Pyridostigmine as first line; may benefit from addition of salbutamol / ephedrine or 3,4-diaminopyridine. Avoid β2-adrenergic agonists (fluoxetine / quinidine)	ORPHA:98913 Postsynaptic congenital myasthenic syndromes	• Fast-channel syndrome, FCS • Kinetic abnormalities of the AChR	
*CHRND*	Congenital myasthenic syndrome due to primary acetylcholine receptor deficiency caused by pathogenic variants in *CHRND*	616323: Myasthenic syndrome, congenital, 3c, associated with acetylcholine receptor deficiency; CMS3c	Pyridostigmine as first line; may benefit from addition of 3,4-diaminopyridine or salbutamol / ephedrine	ORPHA:98913 Postsynaptic congenital myasthenic syndromes	• Primary AChR deficiency	
*CHRND*	Congenital myasthenic syndrome due to defects in acetylcholine receptor clustering caused by pathogenic variants in *CHRND*	N/A	Pyridostigmine	N/A		
*CHRNE*	Slow-channel congenital myasthenic syndrome due to an acetylcholine receptor defect caused by a pathogenic variant in *CHRNE*	605809: Myasthenic syndrome, congenital, 4a, slow-channel; CMS4a Alternative/former titles: Congenital myasthenic syndrome type Ia1, CMS1a1, CMS Ia1	Fluoxetine or quinidine as first line; avoid pyridostigmine / acetylcholinesterase inhibitors	ORPHA:98913 Postsynaptic congenital myasthenic syndromes	• Slow-channel syndrome, SCS • Kinetic abnormalities of the AChR	
*CHRNE*	Fast-channel congenital myasthenic syndrome due to an acetylcholine receptor defect caused by pathogenic variants in *CHRNE*	616324: Myasthenic syndrome, congenital, 4b, fast-channel; CMS4b	Pyridostigmine as first line; may benefit from addition of salbutamol / ephedrine or 3,4-diaminopyridine. Avoid β2-adrenergic agonists (fluoxetine / quinidine)	ORPHA:98913 Postsynaptic congenital myasthenic syndromes	• Fast-channel syndrome, FCS • Kinetic abnormalities of the AChR	
*CHRNE*	Congenital myasthenic syndrome due to primary acetylcholine receptor deficiency caused by pathogenic variants in *CHRNE*	608931: Myasthenic syndrome, congenital, 4c, associated with acetylcholine receptor deficiency; CMS4c Alternative/former titles: Myasthenic syndrome, congenital, type ID; CMS1D, CMS ID, Myasthenia, familial infantile, 1, FIM1,	Pyridostigmine as first line; may benefit from addition of 3,4-diaminopyridine or salbutamol / ephedrine	ORPHA:98913 Postsynaptic congenital myasthenic syndromes	• Primary AChR deficiency	
*CHRNE*	Congenital myasthenic syndrome with kinetic defect due to reduced ion channel conductance caused by pathogenic variants in *CHRNE*	N/A	Pyridostigmine	N/A	• Kinetic abnormalities of the AChR • Reduced ion channel conductance	
*COL13A1*	Congenital myasthenic syndrome due to collagen 13 defects caused by pathogenic variants in *COL13A1*	616720: Myasthenic syndrome, congenital, 19; CMS19	Salbutamol / ephedrine as first line; may benefit from addition of 3,4-diaminopyridine. Pyridostigmine likely ineffective.	ORPHA:98913 Postsynaptic congenital myasthenic syndromes	• Synaptic and basal-lamina associated syndromes • Synaptic space	
*COLQ*	Congenital myasthenic syndrome due to endplate acetylcholinesterase deficiency caused by pathogenic variants in *COLQ*	603034: Myasthenic syndrome, congenital, 5; CMS5 Alternative/former titles: Endplate acetylcholinesterase deficiency; EAD Engel congenital myasthenic syndrome Myasthenic syndrome, congenital, Engel type Congenital myasthenic syndrome type IC, CMS1c, CMS IC	Salbutamol or ephedrine as first line; avoid pyridostigmine / acetylcholinesterase inhibitors	ORPHA:98915 Synaptic congenital myasthenic syndromes	• Synaptic and basal-lamina associated syndromes • Synaptic space	• Endplate AChE deficiency • Endplate acetylcholinesterase deficiency
*DOK7*	Congenital myasthenic syndrome due to defects in docking protein 7 caused by pathogenic variants in *DOK7*	254300: Myasthenic syndrome, congenital, 10; CMS10 Alternative/former titles: Myasthenia, limb-girdle, familial, LGM, Congenital myasthenic syndrome type Ib, CMS1b, CMS Ib, Myasthenic myopathy	Salbutamol or ephedrine as first line; avoid pyridostigmine / acetylcholinesterase inhibitors	ORPHA:98913 Postsynaptic congenital myasthenic syndromes	• Defects within the AChR-clustering pathway • Defects in endplate development and maintenance	• DOK7-associated limb-girdle-myasthenia • DOK7 CMS • Dok-7 myasthenia
*DPAGT1*	Congenital myasthenic syndrome due to a defect of glycosylation caused by pathogenic variants in *DPAGT1*	614750: Myasthenic syndrome, congenital, 13; CMS13 Alternative/former titles: Myasthenic syndrome, congenital, with tubular aggregates 2; CMSta2	Pyridostigmine as first line; may benefit from addition of 3,4-diaminopyridine or salbutamol / ephedrine	N/A	• Limb-girdle-myasthenia with glycosylation deficiency • CMS due to abnormal glycosylation • Congenital defects of glycosylation • Defects in protein glycosylation	• DPAGT1 myasthenia
*GFPT1*	Congenital myasthenic syndrome due to a defect of glycosylation caused by pathogenic variants in *GFPT1*	610542: Myasthenic syndrome, congenital, 12; CMS12 Alternative/former titles: Myasthenic syndrome, congenital, with tubular aggregates 1; CMSTA1	Pyridostigmine as first line; may benefit from addition of 3,4-diaminopyridine or salbutamol / ephedrine	N/A	• Limb-girdle-myasthenia with glycosylation deficiency • CMS due to abnormal glycosylation • Congenital defects of glycosylation • Defects in protein glycosylation	• GFPT1 myasthenia
*GMPPB*	Congenital myasthenic syndrome due to a defect of glycosylation caused by pathogenic variants in *GMPPB*	N/A (615352 is for the LGMD phenotype minus the myasthenic features)	Pyridostigmine as first line; may benefit from addition of 3,4-diaminopyridine or salbutamol / ephedrine	N/A	• Limb-girdle-myasthenia with glycosylation deficiency • CMS due to abnormal glycosylation • Congenital defects of glycosylation • Defects in protein glycosylation	• GMPPB myasthenia
*LAMB2*	Congenital myasthenic syndrome due to laminin beta 2 deficiency caused by pathogenic variants in *LAMB2*	N/A	Salbutamol or ephedrine	ORPHA:98915 Synaptic congenital myasthenic syndromes	• Synaptic basal lamina-associated syndromes	• Laminin beta2 deficiency
*LRP4*	Congenital myasthenic syndrome due to defects in low-density lipoprotein receptor-related protein 4 caused by pathogenic variants in LRP4	616304: Myasthenic syndrome, congenital, 17; CMS17	Salbutamol or ephedrine as first line; avoid pyridostigmine / acetylcholinesterase inhibitors	ORPHA:98913 Postsynaptic congenital myasthenic syndromes	• Defects within the AChR-clustering pathway • Defects in endplate development and maintenance	• LRP4 myasthenia
*MUSK*	Congenital myasthenic syndrome due to defects in MuSK caused by pathogenic variants in MUSK	616,325: Myasthenic syndrome, congenital, 9, associated with acetylcholine receptor deficiency; CMS9	Salbutamol or ephedrine as first line; avoid pyridostigmine / acetylcholinesterase inhibitors	ORPHA:98913 Postsynaptic congenital myasthenic syndromes	• Defects within the AChR-clustering pathway • Defects in endplate development and maintenance	• Congenital MuSK myasthenia • MuSK deficiency
*MYO9A*	Congenital myasthenic syndrome due to a defect in Myosin 9A caused by pathogenic variants in *MYO9A*	N/A	Pyridostigmine	ORPHA:98914 Presynaptic congenital myasthenic syndromes	• Axonal transport • Presynaptic	• Myosin 9a deficiency
*PLEC1*	Congenital myasthenic syndrome due to plectin deficiency caused by pathogenic variants in *PLEC1*	N/A	Pyridostigmine	N/A	• Other myasthenic syndromes	• Plectin deficiency
*PREPL*	Congenital myasthenic syndrome caused by pathogenic variants in *PREPL* that predict reduced filling of synaptic vesicles with ACh	616224: Myasthenic syndrome, congenital, 22; CMS22 Alternative/former titles: PREPL deficiency	Pyridostigmine	N/A	• Limb-girdle-myasthenia with glycosylation deficiency • Synthesis and Recycling of Acetylcholine • Other myasthenic syndromes	• PREPL deletion syndrome • PREPL deficiency
*RAPSN*	Congenital myasthenic syndrome due to endplate rapsyn deficiency caused by pathogenic variants in *RAPSN*	616326: Myasthenic syndrome, congenital, 11, associated with acetylcholine receptor deficiency; CMS11 Alternative/former titles: Myasthenic syndrome, congenital, Ie, CMS1e, CMS Ie	Pyridostigmine as first line; may benefit from addition of 3,4-diaminopyridine or salbutamol / ephedrine	ORPHA:98913 Postsynaptic congenital myasthenic syndromes	• Defects within the AChR-clustering pathway • Defects in endplate development and maintenance	• Endplate rapsyn deficiency • Rapsyn deficiency • Rapsyn CMS
*SCN4A*	Congenital myasthenic syndrome due to a sodium channel 1.4 defect caused by pathogenic variants in *SCN4A*	614198: Myasthenic syndrome, congenital, 16; CMS16 Alternative/former titles: Myasthenic syndrome, congenital, acetazolamide-responsive	Pyridostigmine as first line; acetazolamide may be helpful for periodic paralysis	ORPHA:98913 Postsynaptic congenital myasthenic syndromes	• Other myasthenic syndromes	• Na channel myasthenia • Sodium channel myasthenia
*SLC18A3*	Congenital myasthenic syndrome due to a vesicular acetylcholine transporter defect caused by pathogenic variants in *SLC18A3*	617239: Myasthenic syndrome, congenital, 21, presynaptic; CMS21	Pyridostigmine	ORPHA:98914 Presynaptic congenital myasthenic syndromes	• Synthesis and recycling of acetylcholine	• Vesicular ACh transporter deficiency
*SLC25A1*	Congenital myasthenic syndrome due to a mitochondrial citrate carrier defect caused by pathogenic variants in *SLC25A1*	N/A	Pyridostigmine as first line; may benefit from addition of 3,4-diaminopyridine	ORPHA:98914 Presynaptic congenital myasthenic syndromes	• Other syndromes	• Mitochondrial citrate carrier deficiency
*SLC5A7*	Congenital myasthenic syndrome due to a choline transporter defect caused by pathogenic variants in *SLC5A7*	617143: Myasthenic syndrome, congenital, 20, presynaptic; CMS20	Pyridostigmine as first line; may benefit from addition of salbutamol / ephedrine	ORPHA:98914 Presynaptic congenital myasthenic syndromes	• Synthesis and recycling of acetylcholine	• High-affinity presynaptic choline transporter
*SNAP25B*	Congenital myasthenic syndrome due to a synaptosomal-associated protein 25 defect caused by pathogenic variants in *SNAP25B*	616330: Myasthenic syndrome, congenital, 18; CMS18 Alternative/former titles: Myasthenic syndrome, congenital, 18, with intellectual disability and ataxia	3,4-diaminopyridine	N/A	• Synaptic vesicles exocytosis • Presynaptic	• SNAP25-associated CMS • SNAP25B CMS • SNAP25B deficiency
*SYT2*	Congenital myasthenic syndrome due to a synaptotagmin defect caused by a pathogenic variant in *SYT2*	616040: Myasthenic syndrome, congenital, 7, presynaptic; CMS7 Alternative/former titles: Myasthenic syndrome, presynaptic, congenital, with or without motor neuropathy; MYSPC	3,4-diaminopyridine	ORPHA:98914 Presynaptic congenital myasthenic syndromes	• Synaptic vesicles exocytosis • Presynaptic	• SYT2 CMS • Synaptotagmin 2 myasthenia
*UNC13A*	Congenital myasthenic syndrome due to a mammalian uncoordinated-13 protein defect caused by a pathogenic variant in *UNC13A*	N/A	3,4-diaminopyridine as first line; may benefit from addition of pyridostigmine	N/A	• Synaptic vesicles exocytosis • Presynaptic	• Munc13–1 myasthenia
*VAMP1*	Congenital myasthenic syndrome due to a vesicle associated membrane protein 1 defect caused by a pathogenic variant in *VAMP1*	N/A	Pyridostigmine	N/A	• Synaptic vesicles exocytosis • Presynaptic	• Synaptobrevin-1 myasthenia

**Table 3:** Proposed revision of Orphanet hierarchy below ORPHA:590 (Congenital myasthenic syndrome)

ORPHA number	Typology	Root	Level 1	Level 2	Level 3	Level 4
ORPHA:590	Group of phenomes	Congenital myasthenic syndrome				
ORPHA:98913	Group of phenomes		Postsynaptic congenital myasthenic syndromes			
NEW	Group of phenomes			Congenital myasthenic syndromes with kinetic defect		
NEW	Group of phenomes				Fast-channel congenital myasthenic syndromes	
NEW	Disease					Fast-channel congenital myasthenic syndrome due to an acetylcholine receptor defect caused by a pathogenic variant in *CHRNA1*
NEW	Disease					Fast-channel congenital myasthenic syndrome due to an acetylcholine receptor defect caused by a pathogenic variant in *CHRNB1*
NEW	Disease					Fast-channel congenital myasthenic syndrome due to an acetylcholine receptor defect caused by a pathogenic variant in *CHRND*
NEW	Disease					Fast-channel congenital myasthenic syndrome due to an acetylcholine receptor defect caused by a pathogenic variant in *CHRNE*
NEW	Group of phenomes				Slow-channel congenital myasthenic syndromes	
NEW	Disease					Slow-channel congenital myasthenic syndrome due to an acetylcholine receptor defect caused by a pathogenic variant in *CHRNA1*
NEW	Disease					Slow-channel congenital myasthenic syndrome due to an acetylcholine receptor defect caused by a pathogenic variant in *CHRNB1*
NEW	Disease					Slow-channel congenital myasthenic syndrome due to an acetylcholine receptor defect caused by a pathogenic variant in *CHRND*
NEW	Disease					Slow-channel congenital myasthenic syndrome due to an acetylcholine receptor defect caused by a pathogenic variant in *CHRNE*
NEW	Group of phenomes				Congenital myasthenic syndromes with kinetic defect due to reduced ion channel conductance	
NEW	Disease					Congenital myasthenic syndrome with kinetic defect due to reduced ion channel conductance caused by pathogenic variants in *CHRNE*
NEW	Group of phenomes			Congenital myasthenic syndromes with primary acetylcholine receptor deficiency		
NEW	Disease				Congenital myasthenic syndrome due to primary acetylcholine receptor deficiency caused by pathogenic variants in *CHRNA1*	
NEW	Disease				Congenital myasthenic syndrome due to primary acetylcholine receptor deficiency caused by pathogenic variants in *CHRNB1*	
NEW	Disease				Congenital myasthenic syndrome due to primary acetylcholine receptor deficiency caused by pathogenic variants in *CHRND*	
NEW	Disease				Congenital myasthenic syndrome due to primary acetylcholine receptor deficiency caused by pathogenic variants in *CHRNE*	
NEW	Group of phenomes			Congenital myasthenic syndromes due to primary or secondary defects in acetylcholine receptor clustering		
NEW	Disease				Congenital myasthenic syndrome due to defects in acetylcholine receptor clustering caused by pathogenic variants in *CHRND*	
NEW	Disease				Congenital myasthenic syndrome due to endplate rapsyn deficiency caused by pathogenic variants in *RAPSN*	
NEW	Group of phenomes			Congenital myasthenic syndromes due to defects in endplate development and maintenance		
NEW	Disease				Congenital myasthenic syndrome due to agrin deficiency caused by pathogenic variants in *AGRN*	
NEW	Disease				Congenital myasthenic syndrome due to defects in low-density lipoprotein receptor-related protein 4 caused by pathogenic variants in *LRP4*	
NEW	Disease				Congenital myasthenic syndrome due to defects in muscle-specific kinase caused by pathogenic variants in *MUSK*	
NEW	Disease				Congenital myasthenic syndrome due to defects in docking protein 7 caused by pathogenic variants in *DOK7*	
NEW	Disease			Congenital myasthenic syndrome due to plectin deficiency caused by pathogenic variants in *PLEC1*		
NEW	Disease			Congenital myasthenic syndrome due to a sodium channel 1.4 defect caused by pathogenic variants in *SCN4A*		
ORPHA:98914	Group of phenomes		Presynaptic congenital myasthenic syndromes			
NEW	Group of phenomes			Congenital myasthenic syndromes due to defective axonal transport		
NEW	Disease				Congenital myasthenic syndrome due to a defect in Myosin 9A caused by pathogenic variants in *MYO9A*	
NEW	Group of phenomes			Congenital myasthenic syndromes due to defective synthesis or recycling of acetylcholine		
NEW	Disease				Congenital myasthenic syndrome due to endplate choline acetyltransferase deficiency caused by pathogenic variants in *CHAT*	
NEW	Disease				Congenital myasthenic syndrome caused by pathogenic variants in *PREPL* that predict reduced filling of synaptic vesicles with ACh	
NEW	Disease				Congenital myasthenic syndrome due to a choline transporter defect caused by pathogenic variants in *SLC5A7*	
NEW	Disease				Congenital myasthenic syndrome due to a vesicular acetylcholine transporter defect caused by pathogenic variants in *SLC18A3*	
NEW	Group of phenomes			Congenital myasthenic syndromes due to defective synaptic vesicles exocytosis		
NEW	Disease				Congenital myasthenic syndrome due to a synaptosomal-associated protein 25 defect caused by pathogenic variants in *SNAP25B*	
NEW	Disease				Congenital myasthenic syndrome due to a synaptotagmin defect caused by a pathogenic variant in *SYT2*	
NEW	Disease				Congenital myasthenic syndrome due to a mammalian uncoordinated-13 protein defect caused by a pathogenic variant in *UNC13A*	
NEW	Disease				Congenital myasthenic syndrome due to a vesicle associated membrane protein 1 defect caused by a pathogenic variant in *VAMP1*	
NEW	Disease			Congenital myasthenic syndrome due to a mitochondrial citrate carrier defect caused by pathogenic variants in *SLC25A1*		
ORPHA:98915	Group of phenomes		Synaptic and basal lamina associated congenital myasthenic syndromes^a^			
NEW	Disease				Congenital myasthenic syndrome due to endplate acetylcholinesterase deficiency caused by pathogenic variants in *COLQ*	
NEW	Disease				Congenital myasthenic syndrome due to collagen 13 defects caused by pathogenic variants in *COL13A1*	
NEW	Disease				Congenital myasthenic syndrome due to laminin beta 2 deficiency caused by pathogenic variants in *LAMB2*	
ORPHA:353327	Group of phenomes		Congenital myasthenic syndromes with glycosylation defect			
NEW	Disease			Congenital myasthenic syndrome due to a defect of glycosylation caused by pathogenic variants in *GFPT1*		
NEW	Disease			Congenital myasthenic syndrome due to a defect of glycosylation caused by pathogenic variants in *DPAGT1*		
NEW	Disease			Congenital myasthenic syndrome due to a defect of glycosylation caused by pathogenic variants in *ALG2*		
NEW	Disease			Congenital myasthenic syndrome due to a defect of glycosylation caused by pathogenic variants in *ALG14*		
NEW	Disease			Congenital myasthenic syndrome due to a defect of glycosylation caused by pathogenic variants in *GMPPB*		

^a^name of group updated from “synaptic congenital myasthenic syndromes”

## Results

We defined a total of 39 unique clinical/genetic CMS entities and provided descriptive names for each (Table 2). These were mapped to existing OMIM and Orphanet classifications and existing expert-defined descriptive terms for each were captured from the literature to aid in the definition of group-level classification. Treatment options were obtained from the literature [4, 11] and outlined in Table 2. We then placed the defined entities within the Orphanet classification and hierarchy below the pre-existing entry for congenital myasthenic syndrome, modifying one existing class name and adding 10 group-level phenotypic classes at various levels of the hierarchy and 39 unique disease entities (Table 3).

## Discussion

CMS is classed within the European Union as a rare disease (defined as one that affects fewer than 1 in 2000 individuals) and many of the individual CMS entities are ultra-rare. This has substantial implications for knowledge management, since while much highly expert knowledge on CMS does exist, in common with many other rare diseases this knowledge is often “siloed” in individual research or clinical databases in a few expert centers [12]. Academic publishing still largely relies on “non-machine-readable” formats such as PDF and this again provides a barrier to easy access and reuse [13]. This means that not only do fewer clinicians who encounter CMS patients have the relevant experience themselves, but it is also more challenging and time-consuming for them to locate the information they need.

Clinical, genetic and scientific experts in CMS have come together periodically to review and update classifications of the disease at workshops hosted by the European Neuromuscular Centre [14–16], in NCBI’s GeneReviews series [17] and several comprehensive recent review publications [3, 4, 18]. Broad classifications of CMS into presynaptic, synaptic and postsynaptic CMS and CMS with glycosylation defect were originally proposed in 2001 [15], but it is only with the very latest update to the International Classification of Disease (ICD), Revision 11 [19], that these subgroups even receive a mention (without, however, being allocated a classification number). Meanwhile, as the number and variety of CMS disease entities published in the literature has increased, expert-proposed groupings have been extended to include a new group containing defects of endplate development and maintenance [18]. However, the expert reviews have not attempted any standardization of nomenclature in the coding systems, and at the most granular level, individual “atomic” disease entities or subtypes are conspicuous in their absence from all the coding systems except the Online Mendelian Inheritance in Man (OMIM) database [20]. OMIM itself has good (although not entirely comprehensive) coverage of the individual disease entities, each represented by a “phenotype MIM number” and a sequentially numbered name, and is recognized as the authoritative reference for genetic disorders, but is not itself a nosology or ontology but rather a catalogue, which is thus complementary to (and mappable to) the classification we create here.

To counter the problem of lack of representation of rare disease entities in knowledge systems, bringing data science approaches into the clinical domain has been the focus of a number of recent activities at the European and international level, including the Global Alliance for Genomics and Health (GA4GH) [21], the European Open Science Cloud [22], Big Data to Knowledge (BD2K) [23], the Monarch Initiative [24], GO-FAIR [25], RD-Connect [26] and the new European Joint Programme for Rare Disease to be launched in 2019. Making use of ontologies and coding systems when capturing clinical information and diagnoses is a key step in preparing data for reanalysis and machine-readability [27], but in order for this to be of benefit, the coding system must be fit for purpose – which means it must contain the relevant items in the correct relative positions and at appropriate levels of granularity. If this is not the case, data cannot be appropriately connected or connections may produce misleading results. For example, to a clinician familiar with CMS, it goes without saying that the connection between “congenital myasthenic syndrome” and “responsive to pyridostigmine” is true for CMS caused by *RAPSN* defects and false for that caused by defects in *DOK7*, but a database that only contains an entry for “congenital myasthenic syndrome” has no way of making that distinction. The result of this is that the specific knowledge that is so familiar to the disease experts cannot easily gain wider currency by being made part of online databases or clinical decision support systems, and furthermore the evidence gathered in a clinical setting in support of particular interventions or particular phenotypic associations cannot be fed back into wider practice by from medical or prescribing records, for example.

Of course, no classification in such a rapidly evolving and heterogeneous field can ever be completely comprehensive, and there are always areas where different decisions could be made, such as about the level of granularity or the range of conditions to include. Our inclusion criteria were based primarily on clinical and phenotypic presentation together with some pathomechanistic insights, while a purely gene-based approach might have produced a classification not exclusively including CMS presentations but also kidney or skin disease presentations caused by different defects in the same genes. In addition, there are other neuromuscular conditions that do have detectable morphological and functional disturbances of the neuromuscular junction, but where these are considered to be secondary to the primary pathology or of minor clinical relevance as compared to the primary clinical manifestation (e.g. spinal muscular atrophy or myotubular myopathy). These conditions are classified in different systems and do not appear in our CMS classification. However, from a data science perspective, the choice of what to include or exclude can indeed be left to expert opinion and is of secondary importance compared to the depth and detail of what is covered, and crucially, its internal logic and relationships with other entities and other classification systems [27]. The CMS entities that we have defined fit perfectly as subclasses within the broader coding systems like ICD and SNOMED-CT and map at a 1:1 level to the phenotype MIM numbers where these exist (see Table 2). They can be grouped into preexisting etiological groups such as pre- and post-synaptic (Table 3), and are amenable to multiple other functional, phenotypic and therapeutic groupings as appropriate (“responsive to acetylcholinesterase inhibitors”, “with limb-girdle phenotype”, “associated with episodic apnea” or “characterized by tubular aggregates”, for example).

To take full advantage of the classification developed here, it will be necessary that these next steps are taken, since the development of classification systems, even with names that aim to have some clinical relevance, is of limited diagnostic or therapeutic value in itself. Rather, it should be thought of as the essential foundation onto which more precise clinical and diagnostic pictures of each disease entity can be built, and it is this systematization of knowledge that can then be brought back into the diagnostic and clinical arena to result in improved diagnostic algorithms and clinical information systems. One future development well supported by Orphanet that is a logical extension of the classification to allow improved diagnostic algorithms is the mapping of entities from the classification to their individual phenotypic features using appropriate phenotypic descriptors from ontologies such as the Human Phenotype Ontology [28]. This creates a matrix of detailed information about each disease entity in both computer-accessible and human-readable formats, and is something that can now be achieved for CMS by a similar consensus process. In addition, since many CMSs are treatable, but the treatment varies by type, we can use the classification to differentiate treatments by type as shown in Table 3 and also now have the opportunity to take this further in a machine-readable manner through the development of pharmacogenomic algorithms that give clinicians easier access to specific treatment recommendations once a particular CMS type has been identified. Furthermore, although NGS techniques have still not solved every CMS case, as science advances, we can expect that new genetic defects will be uncovered that account for some of the remaining undiagnosed congenital myasthenic syndromes, and we have thus ensured that this present classification can easily be extended with new entities.

## Conclusions

Knowledge about the full range, etiology and heterogeneity of the congenital myasthenic syndromes has increased rapidly in the NGS era. These diseases present specific challenges owing to their rarity and heterogeneity but also possess certain features – not the least of which is responsiveness to treatment – that make their unambiguous differentiation worthwhile. The benefits of developing a fully granular classification for this group of conditions are thus not purely academic. Although not designed as a diagnostic tool, the detailed classification in a single system of each individual CMS with a defect of neuromuscular transmission as the primary feature provides clinicians and geneticists with an overview of the currently recognized congenital myasthenic syndromes both as individual entities and as logical groupings and this can provide guidance towards the differential diagnoses for a patient with a broad CMS phenotypic presentation. Making use of an unambiguous clinically understandable descriptive name assists in the clinical differentiation of the different diseases, particularly by clinicians less familiar with these rare conditions, while attaching the descriptive name to a code within a recognized coding system enables existing knowledge to be better systematized, thus paving the way towards computer-aided clinical systems and machine-learning algorithms suitable for the NGS era. Through this collaboration between clinical experts and data science experts, we have shown that data science approaches can be used effectively in the clinical domain in a way that does not disrupt preexisting classification by experts and that enhances the utility of preexisting coding systems, building on both to create a more comprehensive result. The classification we have defined can be used in clinical administration systems as an integral part of the Orphanet nomenclature and can be used in scientific publications and clinical case reports to unambiguously define the CMS type in question. It can be extended and modified as required by future scientific advances, but already provides the starting point for the creation of FAIR knowledge bases of data related to the congenital myasthenic syndromes.

## Abbreviations

BD2K: Big Data to Knowledge
CMS: Congenital myasthenic syndrome
FAIR: Findable, accessible, interoperable and reusable
GA4GH: Global Alliance for Genomics and Health
NGS: Next-generation sequencing
NMJ: Neuromuscular junction
OMIM: Online Mendelian Inheritance in Man
